# Constitution of the National Association of Dental Examiners

**Published:** 1890-11

**Authors:** 


### Constitution of The National Association of Dental Examiners, Organized August 6th, 1883.

CONSTITUTION. ARTICLE I. (Name).

This organization shall be known by the name of The National Association of Dental Examiners.

ARTICLE II. (Object).

The objects of this Association shall be to secure, through the operation of the various State Examining Boards a high and uniform standard of qualification for dental practitioners, and so far as practicable, uniformity of methods in the working of these Boards, and of legislation in creating them.

ARTICLE III. (Members).

This Association shall consist of such different State Boards of Dental Examiners as may elect to join this National Association. They may be represented either by a delegate or delegates duly authorized, or by the whole Board. Certificates from the proper officers of any Board will be necessary to entitle such Board to representation in this body.

ARTICLE IV. (Votes).

Each State Board shall be entitled to ten votes. If, at any meeting of this Association but one member of any Board be present, he shall cast the whole number. In case there are more than one member present the ten votes of that Board shall be equally distributed among, and cast by those members of said Board who are present.

ARTICLE V. (Dues).

*Amended April* 2, 1885.

Each State Board becoming connected with this Association shall pay to the Treasurer the sum of five dollars. Assessments, not to exceed five dollars, shall be made annually in case of necessity.

ARTICLE VI. (Officers).

The officers of this Association shall be a President, a Vice-President, and a Secretary and Treasurer, the last named two offices combined in one. They shall be elected by ballot, without nomination, and shall hold their appointments for one year, or until their successors are elected and qualified. A majority of all the votes cast shall be necessary to a choice.

ARTICLE VII. (Duties of Officers).

The President shall preside at all meetings, according to parliamentary usages laid down in "Cushing's Manual." The Vice-President shall perform the duties of the President in case of the latter's absence or inability. The Secretary and Treasurer shall keep correct minutes of the proceedings; give due notice of meetings, and attend to the necessary correspondence. He shall receive and hold all moneys belonging to the Association, and from them shall pay all drafts of the President, countersigned by the Secretary. His accounts shall be audited by a committee of three, appointed annually for that purpose.

ARTICLE VIII. (Obligations of Members).

All State Boards belonging to this Association shall be bound by its action so long as they continue members of it.

Any Board refusing to be bound by the action of this body shall from that time cease to be a member thereof.

ARTICLE IX. (Quorum).

The representatives of five State Boards shall constitute a quorum for the transaction of business.

ARTICLE X. (Meetings).

There shall be held annually a meeting of this Association at such time and place as the Association may determine.

The President may call a meeting at any time during the year upon the written request of five State Boards.

ARTICLE XI. (Amendments).

Amendments to this Constitution may be made at any annual meeting by the consent of all the members present. In case of any opposition, notification in writing shall be made of any such proposed change and shall be laid over for one year for final action, when the amendment can only be adopted by an affirmative vote of three-fourth of the votes present.

NIAGARA FALLS, N. Y., AUGUST 6TH, 1883.

President, J. Taft, Cincinnati, O.; Vice President, G. W.

McElhaney, Columbus, Ga.; Secretary and Treasurer, Geo. H. Cushing, Chicago, Ill.

SARATOGA SPRINGS, N. Y., AUGUST, 8TH, 1884.

President, J. Taft, Cincinnati, O.; Vice-President, G. W. McElhaney, Columbus, Ga.; Secretary and Treasurer, Geo. H. Cushing, Chicago, Ill.

MINNEAPOLIS, MINN., AUGUST 4TH, 1885.

President, J. Taft, Cincinnati, O.; Vice-President, T. S. Waters, Baltimore, Md.; Secretary and Treasurer, Geo. H. Cushing, Chicago, Ill.

NIAGARA FALLS, N.Y., AUGUST 20TH, 1886.

President, J. Taft, Cincinnati, O.; Vice-President, H. A. Smith, Cincinnati, O.; Secretary and Treasurer, Fred. A. Levy, Orange, N. J.

NIAGARA FALLS, N. Y., AUGUST 1ST, 1887.

President, Geo. H. Cushing, Chicago, Ill.; Vice-President, T. S. Waters, Baltimore, Md.; Secretary and Treasurer, Fred. A. Levy, Orange, N. J.

LOUISVILLE, KY., AUGUST, 27TH, 1888.

President, T. S. Waters, Baltimore, Md.; Vice-President, S. T. Kirk, Kokomo, Ind.; Secretary and Treasurer, Fred. A. Levy, Orange, N. J.

SARATOGA SPRINGS, N. Y., AUGUST 6TH, 1889.

President, T. S. Waters, Baltimore, Md.; Vice-President, S. T. Kirk, Kokomo, Ind.; Secretary and Treasurer, Fred. A. Levy, Orange, N. J.

EXCELSIOR SPRINGS, MO., AUGUST 4TH, 1890.

President, T. S. Waters, Baltimore, Md.; Vice-President, C. R. E. Koch, Chicago, Ill.; Secretary and Treasurer, Fred. A. Levy, Orange, N. J.

---

MRS. EMMA EAMES CHASE, of St. Louis, and Jessie M. Ritchy, of Des Moines, Iowa, were admitted to membership in the American Dental Association, at the recent meeting at Excelsior Springs, Mo.

